# Rancher-reported efficacy of lethal and non-lethal livestock predation mitigation strategies for a suite of carnivores

**DOI:** 10.1038/s41598-017-14462-1

**Published:** 2017-10-26

**Authors:** J. D. Scasta, B. Stam, J. L. Windh

**Affiliations:** 1 Department of Ecosystem Science and Management, University of Wyoming, Laramie, WY 82071 USA; 2 Extension, University of Wyoming, Thermopolis, WY USA

## Abstract

Pastoralists have dealt with livestock losses from predators for millennia, yet effective mitigation strategies that balance wildlife conservation and sustainable agriculture are still needed today. In Wyoming, USA, 274 ranchers responded to a retrospective survey, and rated the efficacy of predation mitigation strategies for foxes, dogs, coyotes, wolves, bobcats, mountain lions, bears, and birds (buzzards, eagles, hawks, ravens). Rancher reported efficacy of mitigation varied by predator species, mitigation strategy, and lethality of strategies, but not livestock type. Ranchers perceive they were most effective at mitigating predation by foxes and coyotes, moderately effective at mitigating large carnivores, and the least effective at mitigating birds. Ranchers also reported that avian predators seem to be the most challenging predator type. The general perception was lethal mitigation strategies were more effective than non-lethal strategies, with guard animals showing the most potential among the non-lethal options. In general, ranchers did not perceive non-lethal strategies as a proxy for lethal strategies. However, a few ranchers reported being successful with non-lethal options such as herding, fencing, and stalling at night but more details about such successful applications are needed. Innovation in current or novel non-lethal mitigation strategies, and examples of efficacy, are needed to justify producer adoption.

## Introduction

The interaction between predatory wildlife species, humans, and livestock has been a source of conflict dating back to antiquity and continues to persist in the 21^st^ century^1,2^. Globally, the threat predators pose to economic and food security have contributed to the persistence of this conflict between humans and carnivores^3^. Subsequently, humans have used a variety of strategies to reduce or eliminate predation including guard dogs and shooting^4,5^. In the United States (US), the control efforts were so effective that some predator species were nearly eradicated and protection under the Endangered Species Act (ESA) was initiated for species such as grizzly bears (*Ursus arctos*) in 1975^6^ and gray wolves (*Canis lupus*) in 1978^7^. These conservation strategies limited the lethal management of predators to a point where some species have recovered (Greater Yellowstone distinct population of grizzly bears delisted in 2017^8^ due to recovery; gray wolves delisted nationally in 2013 with the exception of Wyoming which was delisted in 2017^9^). Thus, while the historical human-predator paradigm has shifted from control to coexistence for some predators^10^, this paradigm shift has not occurred for all predators in all places and the complexity of livestock predation persists today^11^.

Currently the conflict between livestock agriculture and predator conservation continues with livestock producers bearing a substantial portion of the cost of growing predator populations through the provision of livestock as an important source of prey^12^. The most current federal surveys estimate that 180,000 sheep were lost to predators at a cost of more than $20 M USD in 2010 and 219,000 cattle were lost to predators at a cost of more than $98 M USD – in the US^13,14^. The cost to the entire industry is low, < 0.01% of the annual gross income of ranches in the northwestern US due to wolf losses for example, but the cost to individually affected ranches is typically much higher because predation is not equitably spread across the entire livestock industry^12^. While predators typically kill out of hunger, it has been documented that excessive killing does occur which further exacerbates the negative connotations ranchers hold for predators^15,16^. Predators are also documented to shift dietary preferences to include livestock prey (as opposed to native prey) and to shift activity and movements according to livestock prey availability and distribution^17^. A commonly used tool for alleviating the economic loss is compensation for kills^18^, yet the current US compensation program may not be applied for all predator species, may ignore the indirect costs of predators, and undercompensate ranchers for the role they have in the conservation of predators^19^ – three issues that are difficult to quantify and assign values to.

Tools available to alleviate predator-caused economic losses, other than compensation from the government for loss, is to avoid livestock predation altogether. Some tools available are non-lethal strategies such as fencing, herding, stalling at night, and guard dogs^20,21^. Other tools available are lethal strategies such as trapping, snaring, and shooting^22^. A recent meta-analysis suggested a general lack of scientific evidence of mitigation strategies reducing the risk of large carnivore predation^23^. Specifically, this review of the literature concluded that some methods do reduce risk of predation, that some methods do not reduce risk of predation, and that there is a high degree of variation within the application of some individual methods such as enclosure, livestock guard dogs, and visual/auditory deterrents^23^.

The debate about the efficacy of managing wildlife predators to save livestock is hindered by a lack of empirical studies of acceptable scientific standards^24^. In the US, subsidized efforts to manage predation on sheep has also been suggested to not result in a stable sheep industry and continued subsidies criticized on the notion that they are simply killing carnivores^25^. Moreover, effective mitigation strategies that balance wildlife conservation and sustainable agriculture are needed^26^. For example, potential elimination of lethal control tactics in a “predator-friendly” approach to allow for the stabilization of dingo (*Canis dingo*) populations have been effective in reducing livestock predation in an Australian case study^27^. Scientists are suggesting the need for this type of paradigm shift from predator control to include asset management that considers the importance of intact wildlife assemblages^28^. Ecologists in particular, are calling for policy makers to eliminate predator control efforts that are lacking empirical data quantifying efficacy and advocating for a higher level of scientific rigor for approval of mitigation methods^29^. There has also been a shift in the general public’s attitude about predator management, in particular more of a preference for “more humane” non-lethal methods. However, the general public does support management of predators when agricultural damage is occurring^30^. Academic wildlife groups have also proposed that non-lethal methods may also be more defensible on ecological, legal, and policy grounds^10^.

Due to (1) the socio-economic and ecological importance of this livestock-predator conflict issue, (2) the lack of empirical assessments of the efficacy of lethal and non-lethal methods, and (3) the advocacy by some to eliminate predator control programs, we surveyed ranchers in a retrospective assessment of experiences with a wide range of mitigation strategies across a suite of predatory wildlife species including: avian predators (buzzards (*Cathartes aura*), eagles (several species), hawks (several species), and ravens (*Corvus corax*)); canid predators (fox (gray fox (*Urocyon cinereoargenteus*), red fox (*Vulpes vulpes*), swift fox (*Vulpes velox*)), coyote (*Canis latrans*), dog (*Canis familiaris*), and wolf (*Canis lupus*)), felid predators (bobcat (*Lynx rufus*) and mountain lion (*Puma concolor*)), and ursid predators (black bear (*Ursus americanus*) and grizzly bear (*Ursus arctos*)). Our objectives were to determine how ranchers in Wyoming, USA, a state with a broad suite of carnivores, rate the efficacy of 8 lethal and non-lethal mitigation strategies and to determine if ranchers perceive non-lethal strategies to be analogous to lethal strategies.

## Results

### Rancher Response

A total of 274 ranchers responded to our 1,046 surveys for a response rate of 26.2%. In the past year, these ranchers lost a total of 25 bulls/cows, 656 calves, 913 rams/ewes, and 3,882 lambs, for a total of 5,476 animals killed by predators. The most lethal predator was coyotes (reported 2,867 individual livestock lost), followed by birds (reported 1,034 individual livestock lost), followed by grizzly bears and then wolves (reported 665 and 582 individual livestock lost respectively) (Table 1). Wolves were the worst predator for bulls/cows (44 mortalities) and coyotes were the worst predator for calves, rams/ewes, and lambs (354 mortalities, 429 mortalities, and 2,079 mortalities respectively) (Table 1).

**Table 1:** Rancher reported livestock mortality by predator type, predator species or species suite, and livestock species and class from 274 ranches in Wyoming for the year prior to the survey.

Type	Predator species or suite of species	Bulls/Cows	Calves	Rams/Ewes	Lambs	Predator Total
Avian	Birds	0	63	61	910	1,034
Canid	Foxes	0	2	0	232	234
	Dogs	2	6	17	7	32
	Coyotes	5	354	429	2,079	2,867
	Wolves	44	186	229	123	582
Felid	Bobcats	0	0	8	71	79
	Mountain Lions	1	46	42	175	264
Ursid	Black Bears	5	14	121	161	301
	Grizzly Bears	12	171	235	247	665
Unknown	Unknown	43	231	136	140	550
Livestock Class Total		25	656	913	3,882	5,476

### What matters more, predator species or mitigation strategy?

Ranchers perceived control of some predator species to be significantly easier than others (*p* < 0.0001; Table 2). Specifically, ranchers reported being moderately effective at mitigating predation by foxes, coyotes, bobcats, and dogs. Reported mitigation efficacy of grizzly bears, black bears, wolves, and mountain lions was more difficult with reports generally between moderate and slight. Responses indicate the most difficult predator to control was birds (buzzards, eagles, hawks, ravens) with reported efficacy generally between slight and not effective (Table 3).

**Table 2:** Non-parametric Van der Waerden scores in a one-way ANOVA and Chi-Squared test to assess the probability > Chi-Square at alpha 0.05.

Fixed Effect	Chi-Square	Pr > Chi-Square
Predator Species	36.1076	<0.0001
Livestock Type	2.3136	0.3145
Mitigation Strategy	114.0011	<0.0001
Lethal/Non-Lethal	98.7555	<0.0001

**Table 3:** Mean and standard error of predation mitigation efficacy across all mitigation strategies for (A) 9 predator species, (B) 8 mitigation strategies, and (C) lethality of mitigation strategies for lethal (4 strategies including shooting, government trappers, private trappers, trapping/snaring) or non-lethal (4 strategies including guard animals, stalling at night, herding, fencing).

Effect Type	Effect	Mean Efficacy Rating*	SE
(A) Predator Species	Fox	2.1^a^	0.2
	Coyote	2.1^a^	0.1
	Bobcat	2.4^ab^	0.2
	Dog	2.5^ab^	0.2
	Grizzly Bear	2.6^b^	0.2
	Black Bear	2.7^b^	0.2
	Wolf	2.7^b^	0.2
	Lion	2.7^b^	0.2
	Birds	3.3^c^	0.1
(B) Mitigation Strategy	Shooting	1.8^a^	0.1
	Trapper_Government	1.9^a^	0.1
	Trapper_Private	2.0^a^	0.1
	Trapping/Snaring	2.0^a^	0.1
	Guard Animal	2.7^b^	0.1
	Stalling at Night	3.1^c^	0.1
	Herding	3.3^c^	0.1
	Fencing	3.6^d^	0.1
(C) Lethality	Lethal	1.9^a^	0.1
	Non-Lethal	3.2^b^	0.1

*Different letters indicate the mean efficacy rating is different at alpha 0.05 and are interpreted within each of the types of effects.

Certain mitigation strategies were reportedly more effective than others (Table 2). Specifically, the lethal methods (shooting, hired trapper, and trapping/snaring) tended to be perceived as the most effective while some of the non-lethal methods were perceived as the least effective with fencing significantly less effective than all mitigation strategies (*p* < 0.0001; Table 3). The non-lethal method reported to have the most potential seems to be the use of guard animals (Table 3). When pooled by lethality, the efficacy of mitigation strategies becomes clear as ranchers, on average, report greater efficacy with lethal than non-lethal methods (Table 2; Table 3). Across all predator species and mitigation strategies and types of strategies, there was no significant difference in reported efficacy by cattle only ranches, sheep only ranches, or ranches with cattle + sheep (*p* = 0.3145; Table 2).

### Avian predators – buzzards, eagles, hawks, and ravens

**Figure 1:**
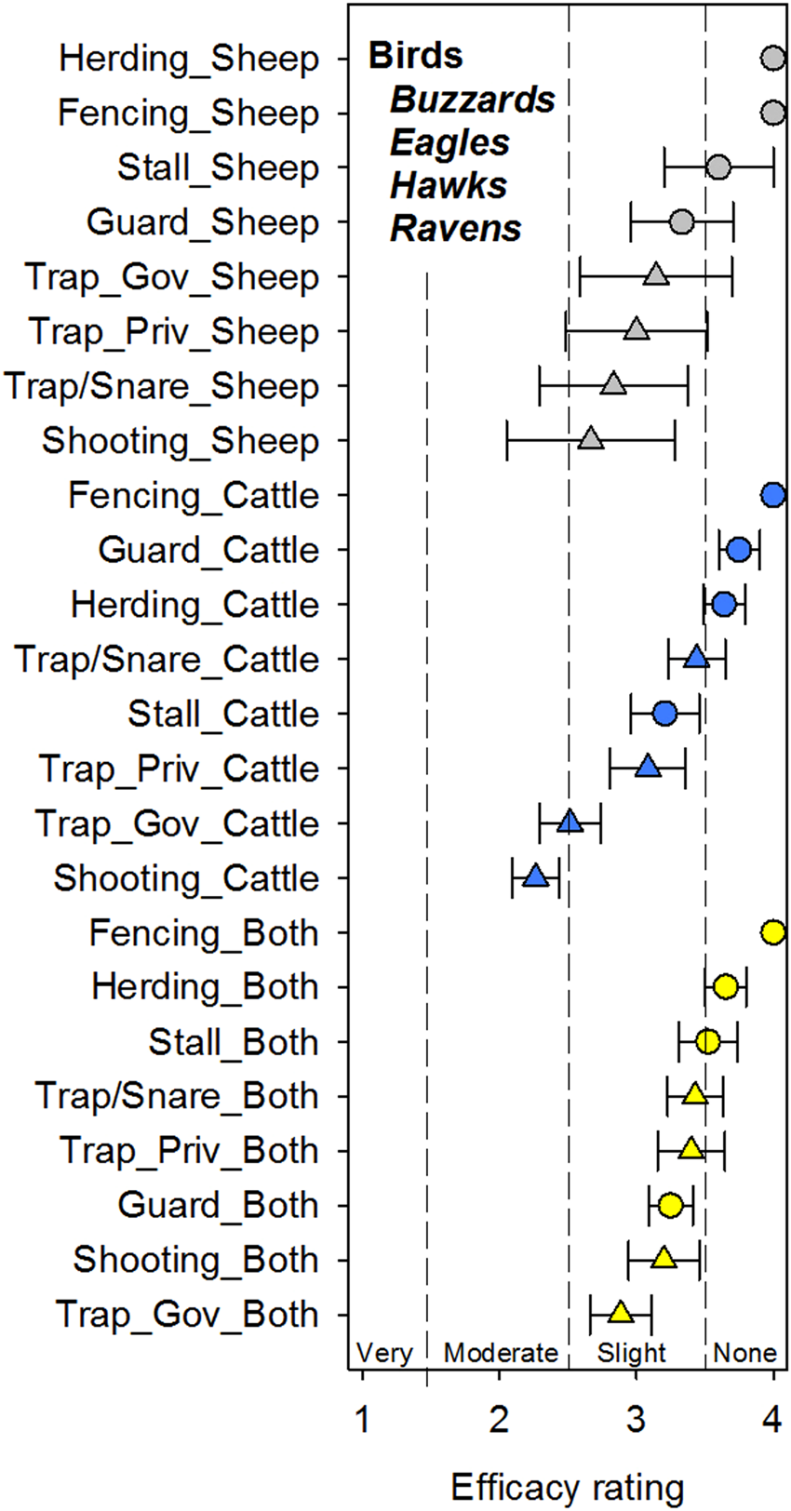
Reported mitigation strategy efficacy mean ± standard error for birds (including buzzards, eagles, hawks, and ravens) on livestock in Wyoming stratified by livestock type (sheep only, cattle only, or cattle + sheep (both)). Mitigation strategies include guard animals (Guard), herding, stalling at night (Stall), shooting, trapping/snaring (Trap/Snare), private trappers (Trap_Priv), and government trappers (Trap_Gov). Non-lethal strategies are denoted by a circle symbol and lethal strategies are denoted by a triangle symbol. For birds, lethality was significant (*p* = 0.0004).

For avian predators regardless of livestock type, ranchers reported that fencing was noted to not be effective unanimously with no error bars due to the consistency of ranchers answering this (which we consider to be an indication of participants paying close attention to the question content) (Fig. 1). Generally, all mitigation strategies, regardless of lethality, were reported by ranchers to be at best slightly effective with the exception of shooting on cattle only operations which was reported as moderately effective (Fig. 1). No methods were considered to be very effective.

### Canid predators – foxes, dogs, coyotes, and wolves

**Figure 2:**
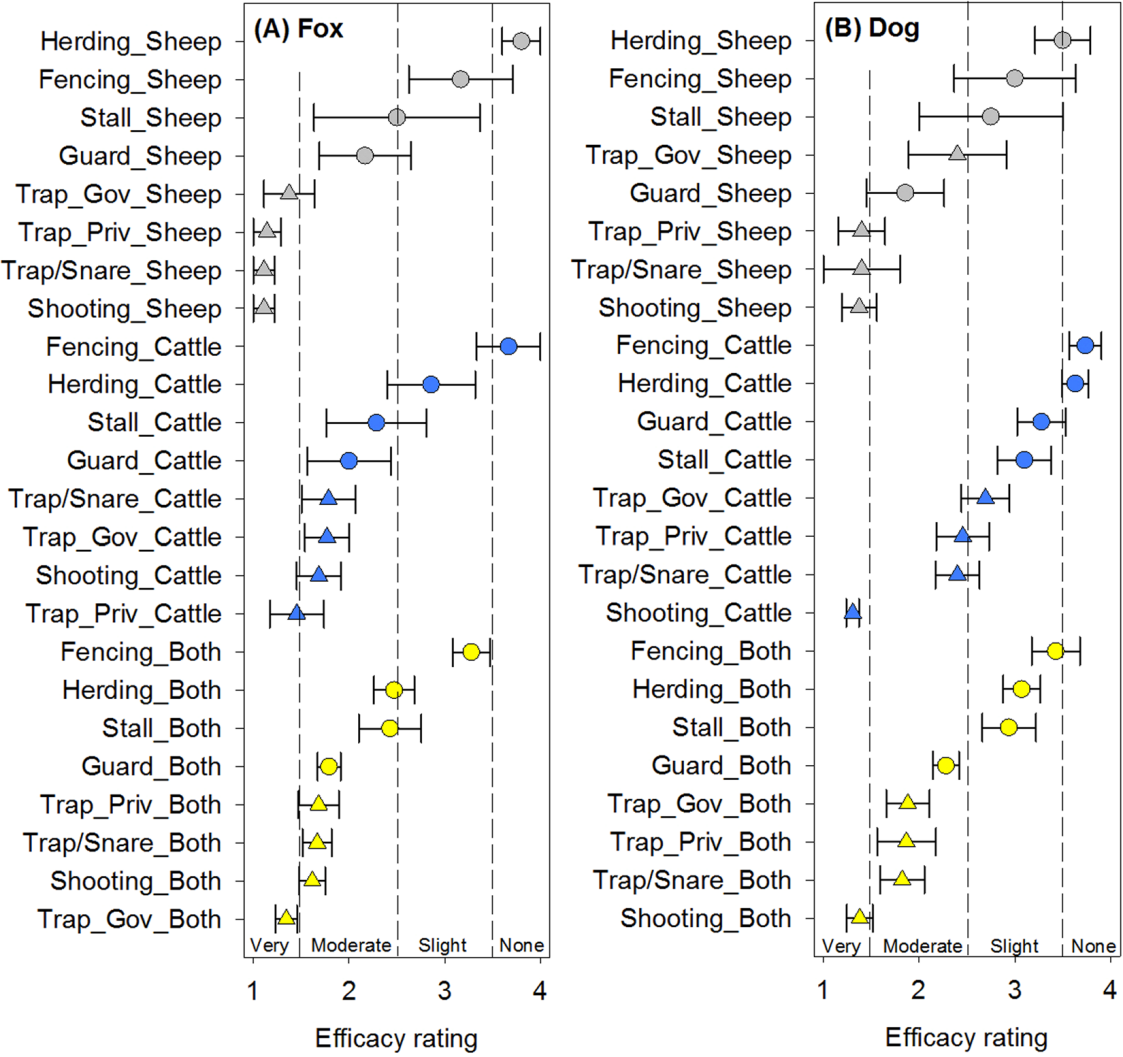
Reported mitigation strategy efficacy mean ± standard error for (**A**) fox and (**B**) dog on livestock in Wyoming stratified by livestock type (sheep only, cattle only, or cattle + sheep (both)). Mitigation strategies include guard animals (Guard), herding, stalling at night (Stall), shooting, trapping/snaring (Trap/Snare), private trappers (Trap_Priv), and government trappers (Trap_Gov). Non-lethal strategies are denoted by a circle symbol and lethal strategies are denoted by a triangle symbol. For foxes and dogs, lethality was significant (*p* < 0.0001 and *p* = 0.0005 respectively).

**Figure 3:**
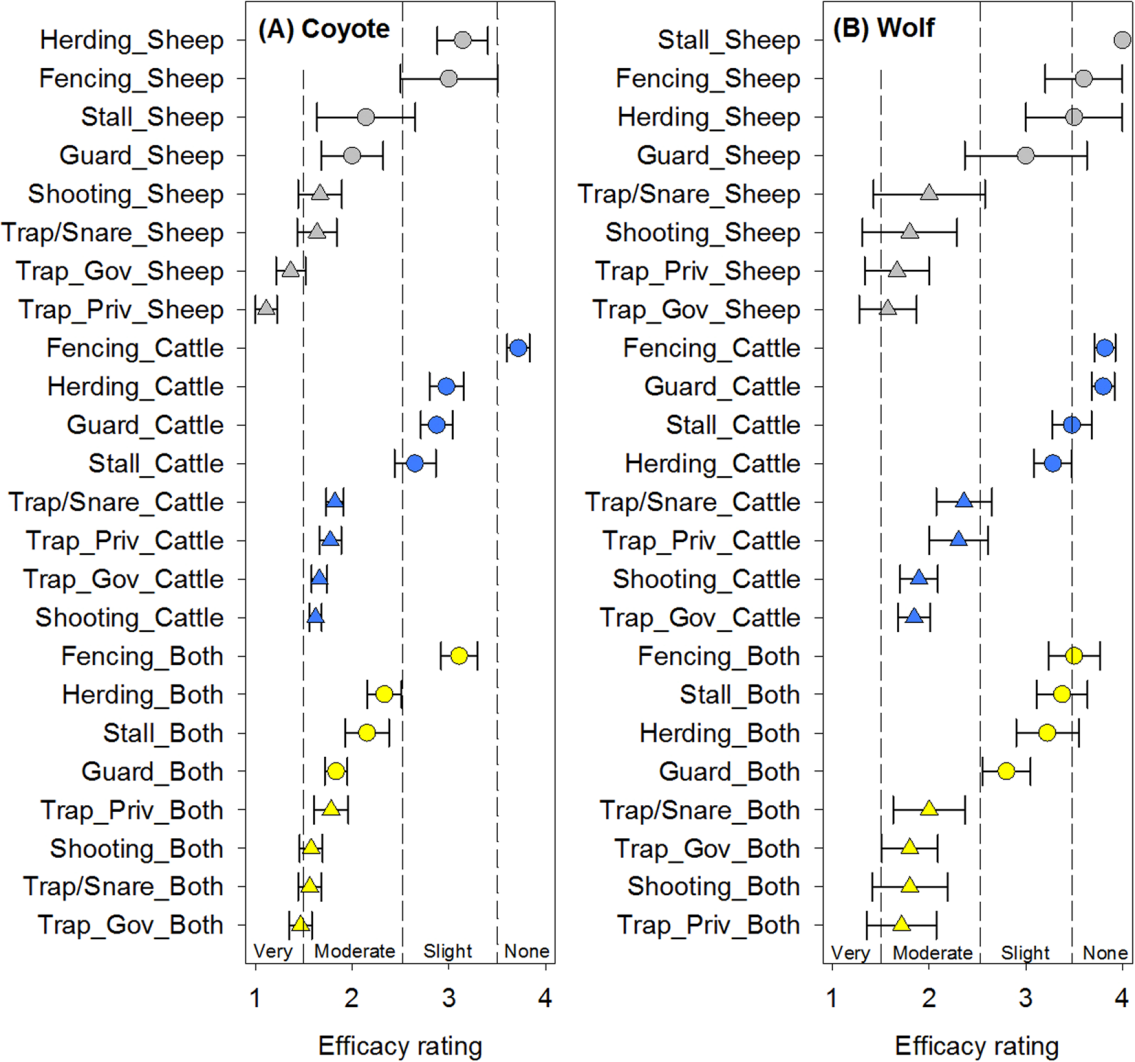
Reported mitigation strategy efficacy mean ± standard error for (**A**) coyote and (**B**) wolf on livestock in Wyoming stratified by livestock type (sheep only, cattle only, or cattle + sheep (both)). Mitigation strategies include guard animals (Guard), herding, stalling at night (Stall), shooting, trapping/snaring (Trap/Snare), private trappers (Trap_Priv), and government trappers (Trap_Gov). Non-lethal strategies are denoted by a circle symbol and lethal strategies are denoted by a triangle symbol. For coyotes and wolves, lethality was significant (*p* < 0.0001 and *p* < 0.0001 respectively).

For foxes, ranchers reported the lethal mitigation strategies were generally very effective and the non-lethal strategies tended to only be moderately or only slightly effective (Fig. 2A). Herding on sheep ranches was generally reported as not effective but the use of guard animals tended to be reported as moderately effective. For cattle operations or operations with cattle + sheep, lethal strategies tended to overlap very and moderate efficacy. For dogs, the lethal mitigation strategies, and in particular shooting, tended to be reported as the most effective especially for operations with cattle. Other lethal options were reported to be effective especially on sheep only operations and the use of guard animals was also reported to be moderate to very effective on sheep only operations (Fig. 2B). For coyotes, ranchers reported that trapping and shooting were the most effective with a noticeable distinction of lethal strategy efficacy on cattle only operations (Fig. 3A). Fencing on cattle only or cattle and sheep operations was reported as slightly to not effective at all. The use of guard animals was reported as moderately effective on sheep or cattle + sheep operations. For wolves, a distinct separation by lethality of mitigation strategies is evident in rancher reports for all livestock types. Generally, the lethal strategies were reported to be moderately effective while the non-lethal strategies were reported to be slightly to not effective (Fig. 3B). In particular, stalling sheep or stalling cattle was reported as not effective. Guard animals were reported to be only slightly effective for sheep, not effective for cattle, and only slightly effective for cattle + sheep operations (Fig. 3B).

### Felid predators – bobcats and mountain lions

**Figure 4:**
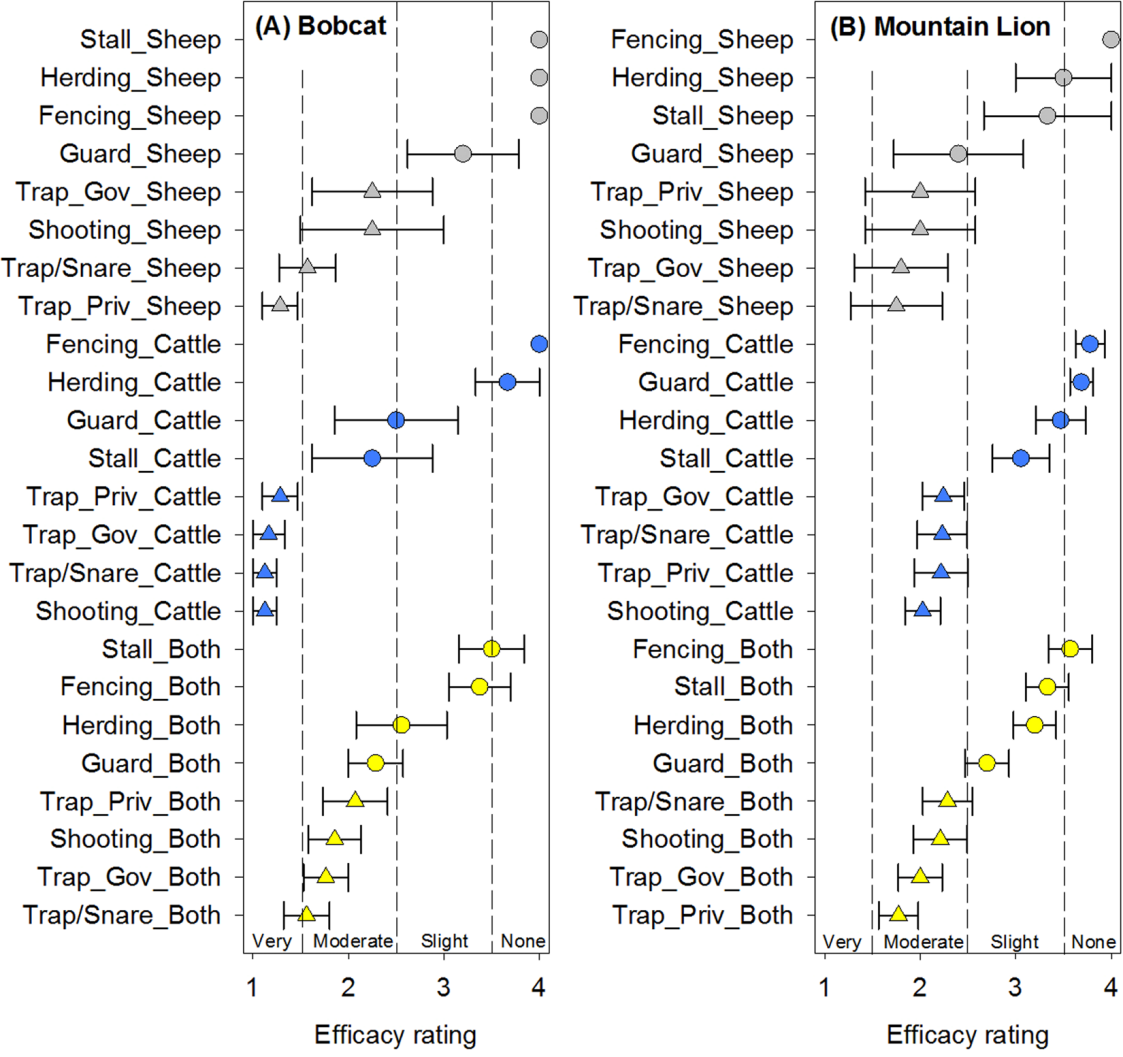
Reported mitigation strategy efficacy mean ± standard error for (**A**) bobcat and (**B**) mountain lion on livestock in Wyoming stratified by livestock type (sheep only, cattle only, or cattle + sheep (both)). Mitigation strategies include guard animals (Guard), herding, stalling at night (Stall), shooting, trapping/snaring (Trap/Snare), private trappers (Trap_Priv), and government trappers (Trap_Gov). Non-lethal strategies are denoted by a circle symbol and lethal strategies are denoted by a triangle symbol. For bobcats and mountain lions, lethality was significant (*p* < 0.0001 and *p* < 0.0001 respectively).

For bobcat mitigation on sheep only operations, stalling, herding, and fencing were all reported to be ineffective and guard animals were reported to only be slightly effective (Fig. 4A). In contrast, the lethal methods were reported to be very or moderately effective. On cattle only operations, all of the lethal options were reported as very effective, stalling and guard animals were reported as moderately effective, fencing and herding were reported as not effective. A similar pattern was displayed on cattle + sheep operations with less efficacy of the lethal mitigation strategies reported by ranchers (Fig. 4A). It is important to note that bobcats were not reported as killing any cattle in our study (Table 1). For mountain lions, a distinct separation by lethality of mitigation strategies is evident according to rancher reports for all livestock types. Generally, the lethal strategies were reported to be moderately effective while the non-lethal strategies were reported to be slightly to not effective with the exception of guard animals for sheep only operations (Fig. 4B). Guard animals were reported to be ineffective for cattle and only slightly effective for cattle + sheep operations (Fig. 4B).

### Ursid predators – black bears and grizzly bears

**Figure 5:**
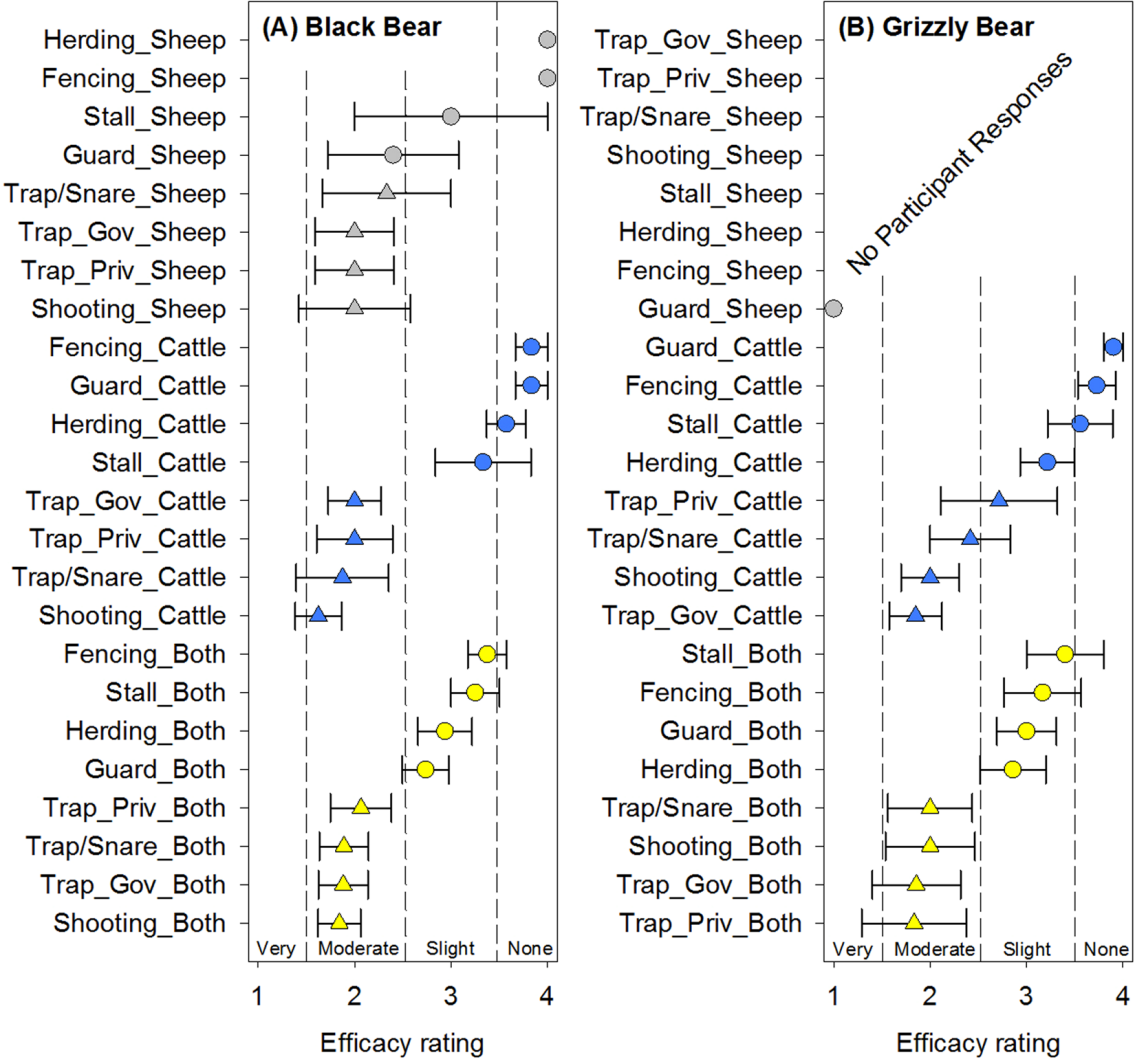
Reported mitigation strategy efficacy mean ± standard error for (**A**) black bear and (**B**) grizzly bear on livestock in Wyoming stratified by livestock type (sheep only, cattle only, or cattle + sheep (both)). Mitigation strategies include guard animals (Guard), herding, stalling at night (Stall), shooting, trapping/snaring (Trap/Snare), private trappers (Trap_Priv), and government trappers (Trap_Gov). Non-lethal strategies are denoted by a circle symbol and lethal strategies are denoted by a triangle symbol. For black bears and grizzly bears, lethality was significant (*p* < 0.0001 and *p* = 0.0149 respectively).

For black bears, herding and fencing on sheep operations were reported as not effective (note *n* = 1 respectively) and stalling sheep elucidated a high level of variability in responses with the mean indicating only slightly effective. The lethal mitigation strategies general were reported as moderately effective regardless of livestock type. One notable exception is that ranchers reported that guard animals on sheep only operations was similar to the lethal mitigation strategies but was less effective on cattle only operations or cattle + sheep operations (Fig. 5A). Non-lethal mitigation strategies on cattle only operations were reported as only slightly or not effective but non-lethal mitigation strategies were generally reported as slightly effective on cattle + sheep operations. For grizzly bears on sheep only operations, there were no rancher responses for any of the lethal mitigation strategies or the non-lethal mitigation strategies with the exception of guard animals (*n* = 1) which was reported as very effective (Fig. 5A). For cattle and cattle + sheep operations, there was a distinct separation by lethality of mitigation strategies evident according to the rancher responses (Fig. 5B). Generally, the lethal strategies were reported to be moderately effective while the non-lethal strategies were reported to be slightly to not effective with the exception of private trappers on cattle only operations (Fig. 5B). Guard animals and fencing were reported to be ineffective for cattle only operations.

### Consider outliers as evidence of potential efficacy and novel applications

The mean and variation around the mean is an indicator of general trends, but based on anecdotal evidence of the efficacy of some non-lethal mitigation strategies, we also consider the minimum or lowest efficacy rating (an indication of potentially very effective application of a mitigation strategy) which are presented in Appendix A and B. For foxes, dogs, and coyotes, almost every non-lethal mitigation strategy, with the exception of herding to mitigate dog predation, had at least one participant reporting a very effective rating. For wolves, every non-lethal mitigation strategy had at least one participant reporting moderate efficacy and, in some cases, at least one participant reported very effective for guard animals, herding, or stalling for one livestock type (the exception being for fencing which was at best only moderately effective). For lions and bobcats, at least one participant reported very effective for each of the non-lethal methods for at least one livestock type (the exception being fencing, herding, or stalling sheep). For both bear species, at least one participant reported very effective for each of the non-lethal methods for at least one livestock type (the exception being fencing for any livestock type). Finally, for avian predators, at least one participant reported guard animals were very effective for each of the livestock types, that stalling at night was very effective for cattle only or cattle + sheep operations, but no participants reported fencing or herding as very effective (Appendix B). In contrast, for all of the lethal mitigation strategies and all of the predator species, at least one participant noted no efficacy for at least one livestock type and frequently for all livestock types (Appendix B). Thus, the variation across rancher responses suggest that lethal and non-lethal mitigation strategies are neither a guaranteed solution or a guarantee for failure depending on finer-scale details than our sampling was able to parse out. Future surveys need to further address this heterogeneity and ask ranchers about their first-hand experience with mitigation strategies and why they were considered a failure or a success.

## Discussion

In our study, ranchers reported that lethal mitigation strategies were more effective than non-lethal mitigation strategies. However, it is important to note that this is an assessment of one stakeholder group within the predator-livestock community and should be interpreted as such. The general consensus among our participants was that fencing, stalling, and herding were mostly not effective to only slightly effective at best, depending on the predator species. This result presents a social difficulty given the greater public preference for non-lethal methods^30^. Currently, it is also difficult then for ranchers to shift from the predator control paradigm to the coexistence paradigm when the suite of non-lethal methods lack the same level of perceived efficacy. In other words, lethal mitigation strategies and non-lethal strategies are not perceived to be analogous by the rancher participants in our study. In particular, this potentially will continue to hinder predator restoration for carnivores to serve their ecological role relative to trophic cascades which is an emerging topic^10,31,32^. However, the use of all non-lethal methods are not without their own collateral damage. For example, extensive fencing can be a form of fragmentation and can hinder migration of native large ungulates and alter predator populations^33,34^.

For livestock production and predator conservation to become more compatible, innovation in the techniques and applications of non-lethal mitigation strategies is needed. The use of guard animals seems to be the most promising non-lethal technique according to our results and as demonstrated for sheep in the US and Africa^20,21^. To enhance efficacy and perceptions of efficacy, it might be important for ranchers to consider other breeds or species of guard animals that would be more aggressive towards predators or capable of physically deterring large carnivores if they are ranching in areas with wolves or bears^35^. It is also possible to learn about how guard animals, in particular guard dogs, have been used in other countries where certain lethal options (such as shooting) are not widely available and the use of guard dogs has been the primary mitigation strategy for many centuries^36^. For example, when the ratio of livestock to guard dog exceeds 100, then efficacy might be reduced^37^. Moreover, effective application of non-lethal strategies in extensive rangeland situations are needed^36^. One other promising application of a non-lethal technique is the use of herding that explicitly integrates low-stress handling techniques and enhances the herd instinct in livestock^38^.

Our results indicate that ranchers perceive mitigating livestock losses due to avian predators to be more difficult than any of the ground-dwelling predator species. This is an important finding in the larger context of human-wildlife interactions as a recent meta-analyses makes no mention of avian predator species^23^. There is also evidence that some avian species such as ravens have been increasing in the past several decades and may also be having a negative effect on native wildlife species such as sage-grouse (*Centrocercus urophasianus*)^39^. In other words, an increasing predatory bird that is protected may be having a detrimental impact on a potentially declining bird that has been petitioned for protection. While the function of avian predators is important, especially in reducing and removing carcasses, balancing livestock production and avian conservation is challenging^40^. Moreover, the four types of avian predators that we asked about all have species protected through the Migratory Bird Treaty Act including buzzards (turkey vulture (*Cathartes aura*), (bald eagle (*Haliaeetus leucocephalus*) and golden eagle (*Aquila chrysaetos*)), hawks (several species including ferruginous (*Buteo regalis*), red-tailed (*Buteo jamaicensis*), and Swainson’s (*Buteo swainsoni*)), and ravens (common raven (*Corvus corax*))^41^. Finally, a persistent challenge for understanding livestock predation by birds is their role as scavengers. It could be difficult to separate the rancher reported livestock losses attributed to predatory birds in our study as killed or scavenged as these birds regularly scavenge carcasses that died from illness or other natural causes and are attracted to calving sites due to afterbirth and neonate availability^42^. However, it is clear that vultures, ravens, and eagles do peck vulnerable soft parts (eyes, rectum, genitals, nose) of neonates and at times adults, are a problem for sheep and cattle in at least 18 US states, known to attack ewes and heifers/cows in parturition, and have been documented injuring and killing calves^43,44^.

## Conclusions

Our retrospective assessment of ranchers operating in an area with a broad suite of avian, canid, felid, and ursid predators indicates the challenge of integrating predator conservation and livestock production with non-lethal mitigation strategies continues to persist. However, it is important to note that this is an assessment of one stakeholder group within the larger stakeholder base, is an indirect assessment using perceptions rather than a direct experimental approach, and should be interpreted as such. Nonetheless, the voices of ranchers are important because they are in the trenches so to speak balancing livestock care with natural resource management. While lethal methods are perceived by ranchers as more effective, in some cases variation of livestock loss is not explained by the control efforts and a more consistent and scientifically based federal predator control program is needed to deal with long-term uncertainties^22^. In addition, innovation in the types of non-lethal mitigation strategies and more details about effective applications of those strategies are needed to enhance and justify rancher adoption. This could include ancient techniques such as the use of guard dogs and/or herding or more contemporary developments in the use of technology. Our results also suggest that it is not the large carnivores that are perceived to be the most difficult to control, but rather it is avian predators. This difficulty is a combination of the aerial access and visual detection that avian predators have and that ground dwelling predators do not, and the restrictions imposed by wildlife protection laws. In other words, it is difficult to protect livestock that are predated by wildlife that are also protected yet have the advantage of flight. For predator conservation and livestock production on extensive rangelands and forest lands of the western US to coexist in a compatible fashion, the voices and experiences of the ranchers in our study should propel the next generation of scientists to integrate old and new technologies to more effectively address this persistent socioecological issue.

## Materials and Methods

### Survey methods

We surveyed ranchers through a cooperative effort by the Wyoming Stock Growers Association (WSGA), Wyoming Wool Growers Association (WWGA), University of Wyoming (UW) Extension, and the Wyoming Department of Agriculture’s Animal Damage Management Board. Protection of human subjects was ensured via approval by the UW Institutional Review Board under protocol #20150922DS00903 and all study methods were performed in accordance with the relevant guidelines and regulations of the approval. There were no risks to participants associated with this project greater than those encountered in daily life. Because the survey was voluntary we informed potential participants that “completion of the survey is consent” which was coupled with a notification letter discussing the purpose of the study, what to expect, risks, benefits, compensation, rights, confidentiality, contacts, and consent. We mailed 816 surveys to WSGA and WWGA members and 230 to UW Extension offices with options to complete the survey in paper or electronic form^45^. Ranchers were asked to rank the efficacy of four non-lethal predator mitigation strategies (guard animals, fencing, herding, stalling at night (i.e., penning, corralling, or confining in an enclosed area)) and four lethal predator mitigation strategies (shooting, trapping/snaring, private trapper, government trapper). Any indication of mitigation method efficacy is assumed to be within the scope of the law and administered under the appropriate permissions or by federal technicians, and an indication first-hand knowledge by the participant. The index for efficacy rating used a four-point scale that estimates the probability or likelihood of efficacy (1 = Very Effective, 2 = Moderately Effective, 3 = Slightly Effective, 4 = Not Effective)^46^ for 9 predator species including: avian predators including a suite of birds specified to participants as buzzards (also known as turkey vultures) (*Cathartes aura*), eagles (several species), hawks (several species), and ravens (*Corvus corax*); four canid species – fox (three species in Wyoming including gray fox (*Urocyon cinereoargenteus*), red fox (*Vulpes vulpes*), swift fox (*Vulpes velox*)), coyote (*Canis latrans*), dog (*Canis familiaris*), and wolf (*Canis lupus*); 2 felid species – bobcat (*Lynx rufus*) and mountain lion (*Puma concolor*); and 2 ursid species – black bear (*Ursus americanus*) and grizzly bear (*Ursus arctos*). Before the efficacy rating answer options, and to ensure results reflect only the experience of participants dealing with a specific predator, participants were asked to indicate if that specific predator was not a problem. The survey was initially administered in April 2016 with the final response collected in April 2017.

### Statistical analyses

Our rating scale is quantifying the likelihood of a mitigation strategy being effective (or not effective) along a continuous, yet simplified cline (ranging from None, Slight, Moderate, and Very) and is asking participants to position themselves at 1 of 4 equidistant anchors^47,48^. We first assessed histograms of frequency distributions of the raw data for each Predator Species*Mitigation Strategy combination and normality of the same raw data combinations using a Shapiro-Wilk normality test^49^. Frequency distribution histograms are provided in Appendix A. We then calculated the mean, standard error, median, and mode of participant efficacy response. These provide an indication of general efficacy and measures of central tendency of participant efficacy ratings to understand that a specific mitigation strategy would or would not be effective. Due to the non-normal data distribution and semi-interval response data, we compared mean efficacy ratings using non-parametric Van der Waerden scores in a one-way ANOVA and Chi-Squared test to assess the probability > Chi-Square at alpha 0.05 for four fixed effects individually: (1) Predator Species, (2) Livestock Type, (3) Mitigation Strategy, and (4) Lethal/Non-Lethal Attribute of Mitigation Strategy^50,51^. For each fixed effect that was statistically significant at alpha 0.05, we then conducted pairwise comparisons within each effect at alpha 0.05. To further understand the responses we then assessed the median, mode, maximum, and minimum for each Livestock Type*Predator Species*Mitigation Strategy combination. By assessing the maximum and minimum efficacy rating we can consider potential situations where individuals have either experienced success or failure with a particular mitigation strategy. Due to the difficulty of presenting such a large data matrices we have placed this data in Appendix B. Figures were prepared in SigmaPlot^49^ and analyses were conducted in SAS with the non-parametric analyses using the npar1way procedure^52^. Raw data is available as a supplement to this manuscript (10.6084/m9.figshare.5508223.v1).

## Electronic supplementary material

Appendix A. Histograms and frequency distributions

Appendix B. Efficacy rating sample size, median, mode, maximum, and minimum stratified by predator species, mitigation strategy, and livestock type.
