# Author Correction: Rancher-reported efficacy of lethal and non-lethal livestock predation mitigation strategies for a suite of carnivores

**DOI:** 10.1038/s41598-018-26270-2

**Published:** 2018-05-16

**Authors:** J. D. Scasta, B. Stam, J. L. Windh

**Affiliations:** 1 Department of Ecosystem Science and Management, University of Wyoming, Laramie, WY 82071 USA; 2 Extension, University of Wyoming, Thermopolis, WY USA

Correction to: *Scientific Reports* 10.1038/s41598-017-14462-1, published online 26 October 2017

The Article contains errors in Table 1 where the reported Livestock Class Totals for each class of livestock are incorrect due to an incorrectly written line of code, which did not include the mortalities from Wolves and Unknown.

The correct totals should read 112, 1073, 1278, 4145 and 6608 respectively.

As a result, in the Results section under the subheading ‘Rancher Response’,

“A total of 274 ranchers responded to our 1,046 surveys for a response rate of 26.2%. In the past year, these ranchers lost a total of 25 bulls/cows, 656 calves, 913 rams/ewes, and 3,882 lambs, for a total of 5,476 animals killed by predators.”

should read:

“A total of 274 ranchers responded to our 1,046 surveys for a response rate of 26.2%. In the past year, these ranchers lost a total of 112 bulls/cows, 1,073 calves, 1,278 rams/ewes, and 4,145 lambs, for a total of 6,608 animals killed by predators.”

