# Supplementary material for: Rancher-reported efficacy of lethal and non-lethal livestock predation mitigation strategies for a suite of carnivores
Source: Sci Rep. 2017 Oct 26;7:14105. doi: 10.1038/s41598-017-14462-1 (PMC5658346; doi:10.1038/s41598-017-14462-1)
Supplement: Supplementary file 1 — Appendix A. Histograms and frequency distributions [file 41598_2017_14462_MOESM1_ESM.pdf]

Rancher-reported efficacy of lethal and non-lethal livestock predation mitigation strategies for a suite of carnivores

J.D. Scasta, B. Stam, and J. L. Windh

Appendix A. Histograms and frequency distributions

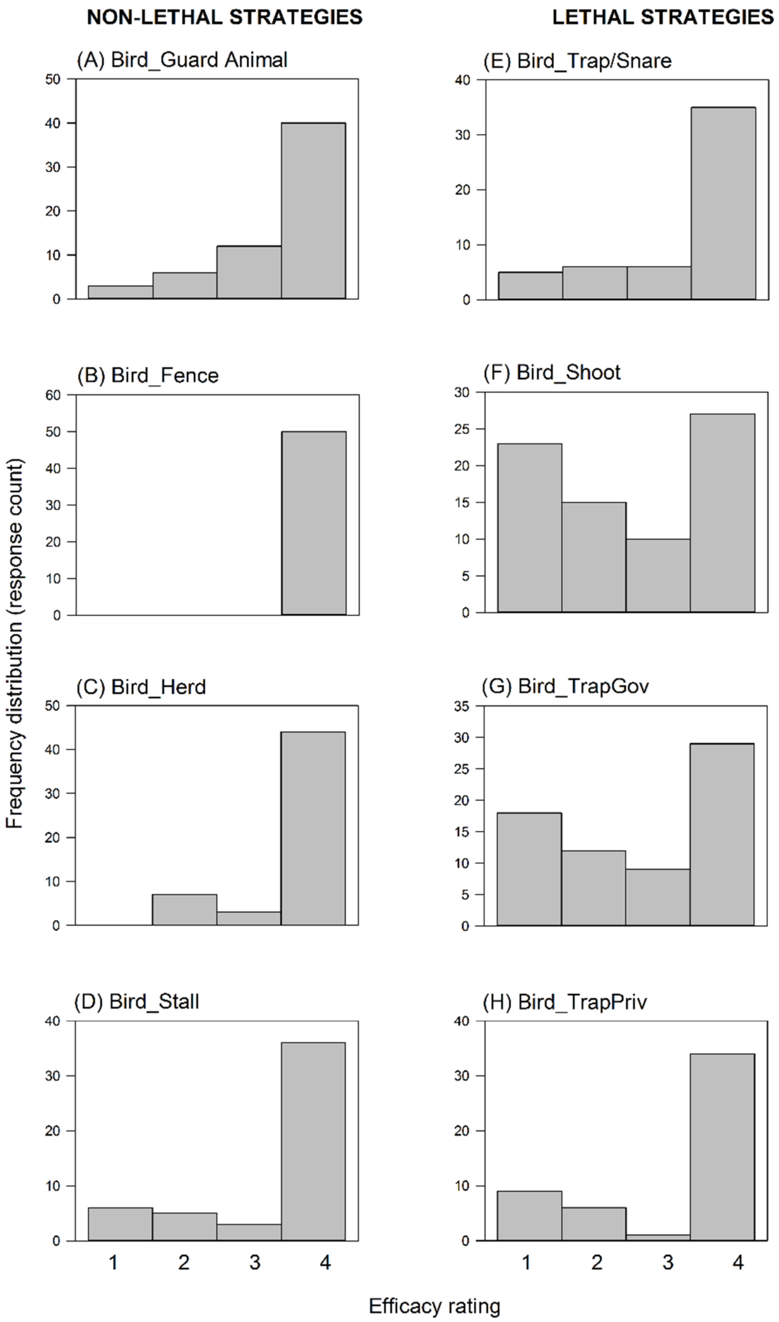

Figure A1. Histogram and frequency distribution of responses indicating efficacy of non-lethal (A – Guard Animals, B – Fencing, C – Herding, D – Stalling) and lethal (E – Trapping/Snaring, F – Shooting, G – Government Trapper, H – Private Trapper) mitigation strategies on birds (buzzards, eagles, hawks, ravens).

Appendix A. Histograms and frequency distributions

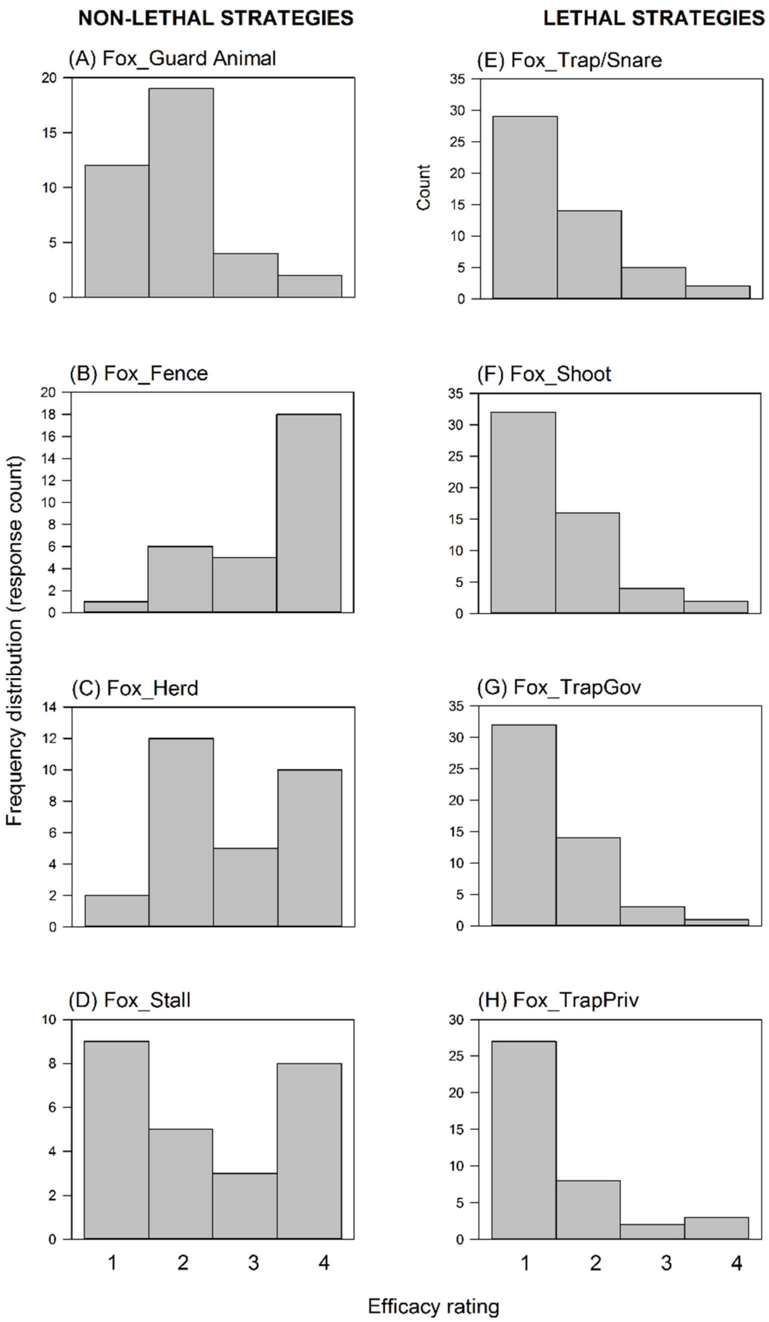

Figure A2. Histogram and frequency distribution of responses indicating efficacy of non-lethal (A – Guard Animals, B – Fencing, C – Herding, D – Stalling) and lethal (E – Trapping/Snaring, F – Shooting, G – Government Trapper, H – Private Trapper) mitigation strategies on foxes.

Appendix A. Histograms and frequency distributions

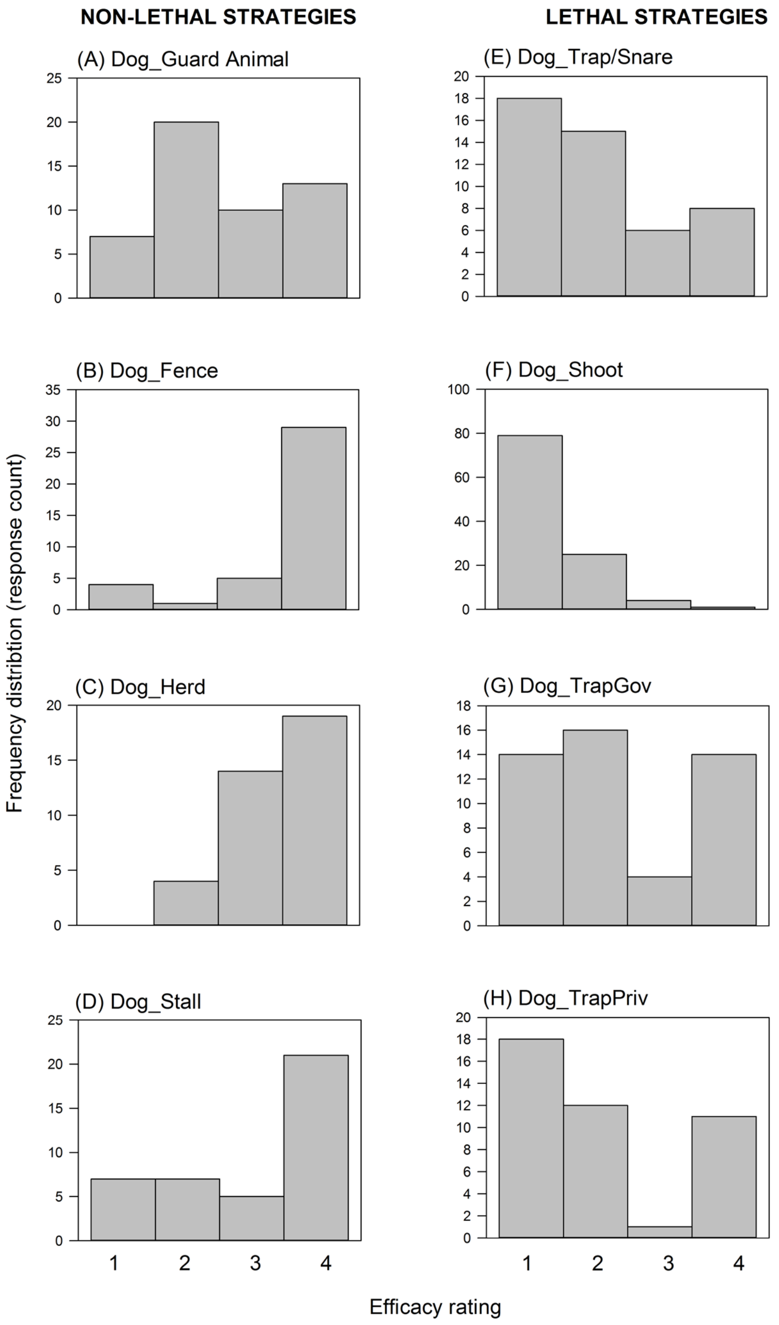

Figure A3. Histogram and frequency distribution of responses indicating efficacy of non-lethal (A – Guard Animals, B – Fencing, C – Herding, D – Stalling) and lethal (E – Trapping/Snaring, F – Shooting, G – Government Trapper, H – Private Trapper) mitigation strategies on dogs.

Appendix A. Histograms and frequency distributions

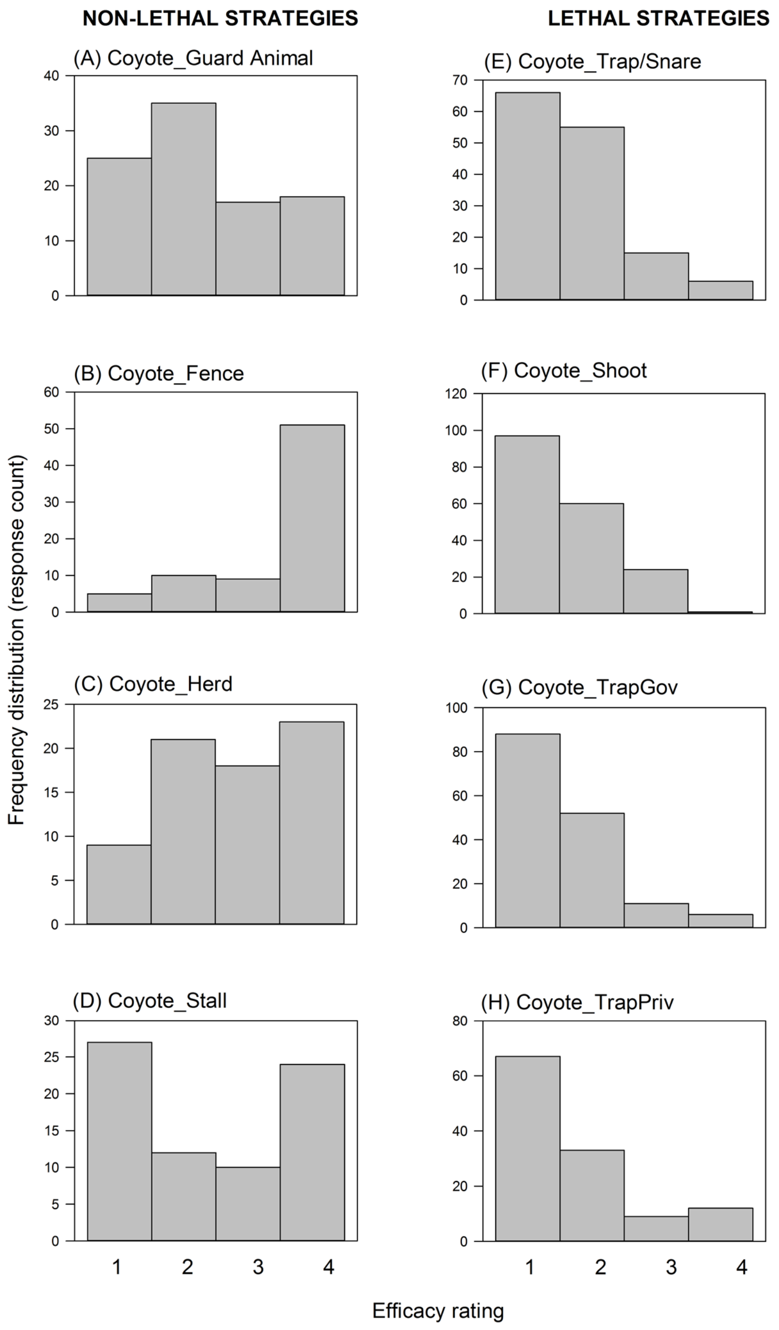

Figure A4. Histogram and frequency distribution of responses indicating efficacy of non-lethal (A – Guard Animals, B – Fencing, C – Herding, D – Stalling) and lethal (E – Trapping/Snaring, F – Shooting, G – Government Trapper, H – Private Trapper) mitigation strategies on coyotes.

Appendix A. Histograms and frequency distributions

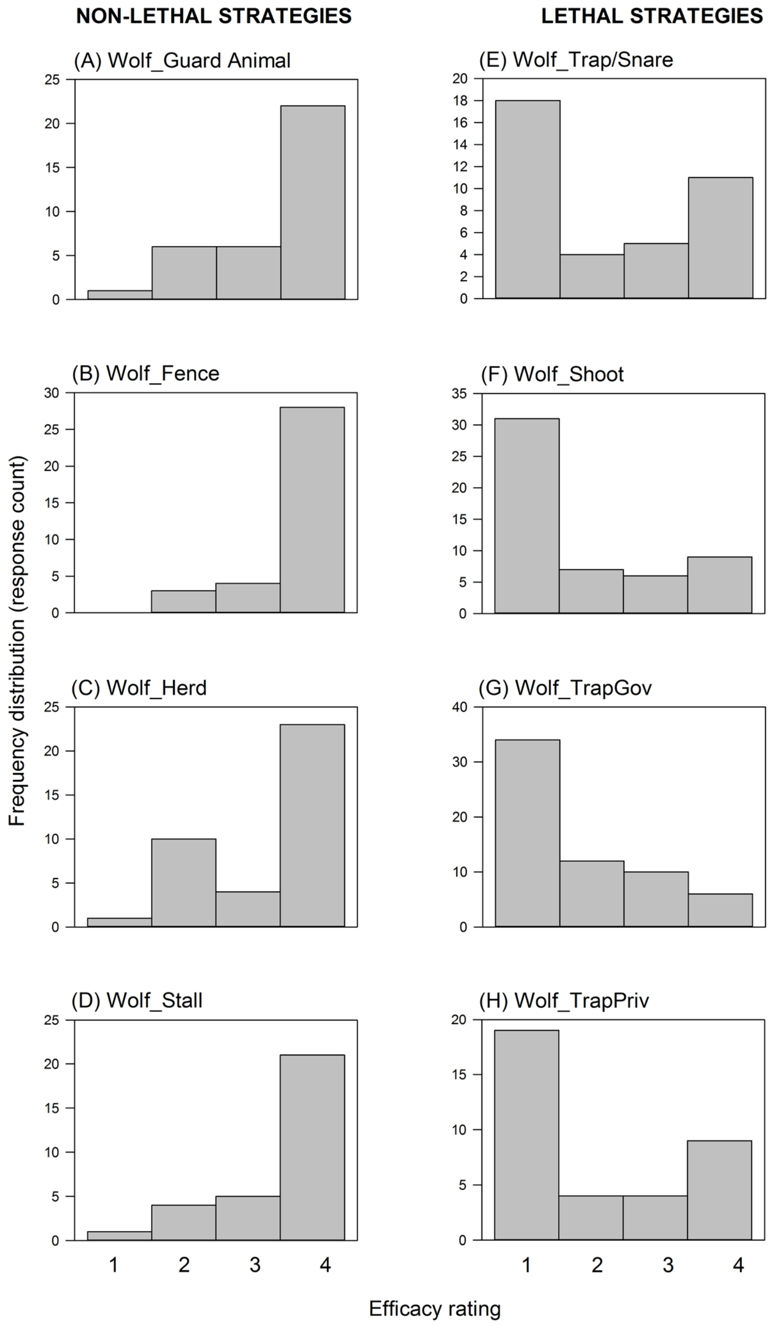

Figure A5. Histogram and frequency distribution of responses indicating efficacy of non-lethal (A – Guard Animals, B – Fencing, C – Herding, D – Stalling) and lethal (E – Trapping/Snaring, F – Shooting, G – Government Trapper, H – Private Trapper) mitigation strategies on wolves.

Appendix A. Histograms and frequency distributions

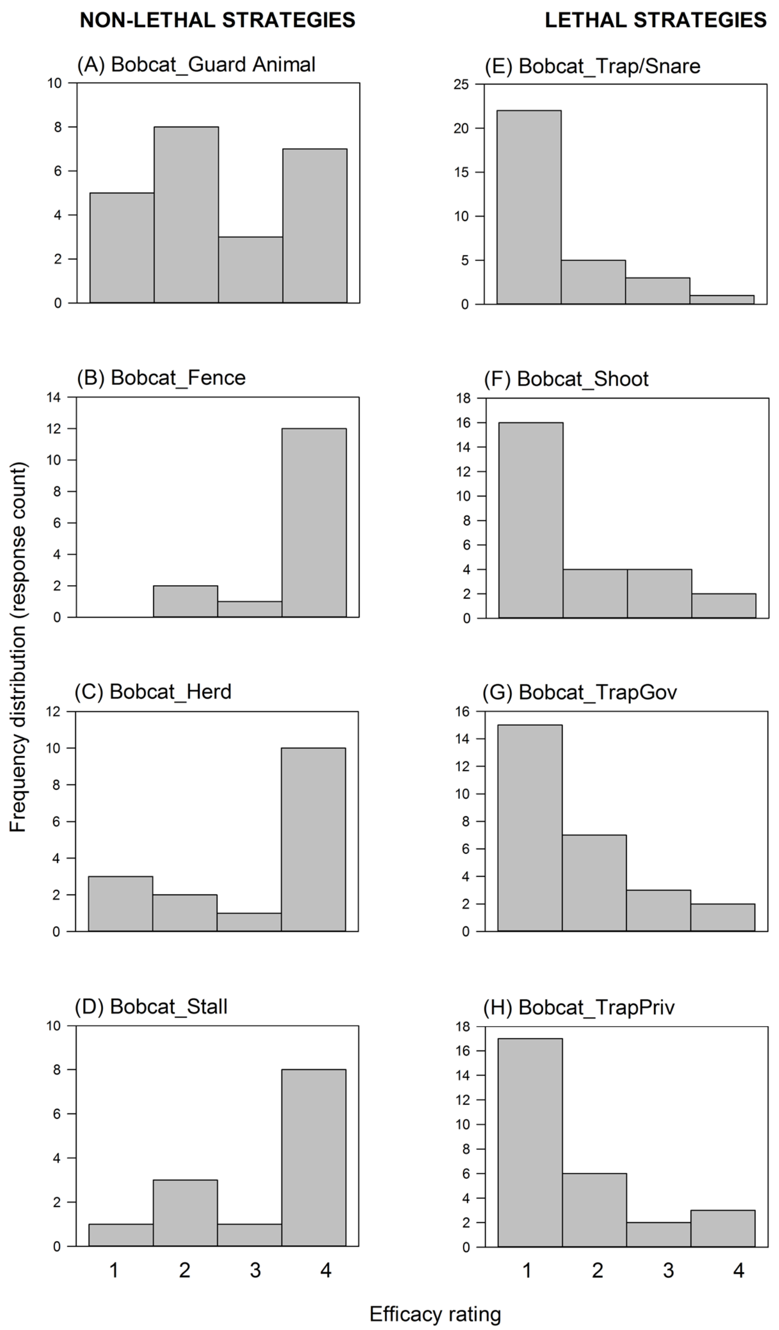

Figure A6. Histogram and frequency distribution of responses indicating efficacy of non-lethal (A – Guard Animals, B – Fencing, C – Herding, D – Stalling) and lethal (E – Trapping/Snaring, F – Shooting, G – Government Trapper, H – Private Trapper) mitigation strategies on bobcats.

Appendix A. Histograms and frequency distributions

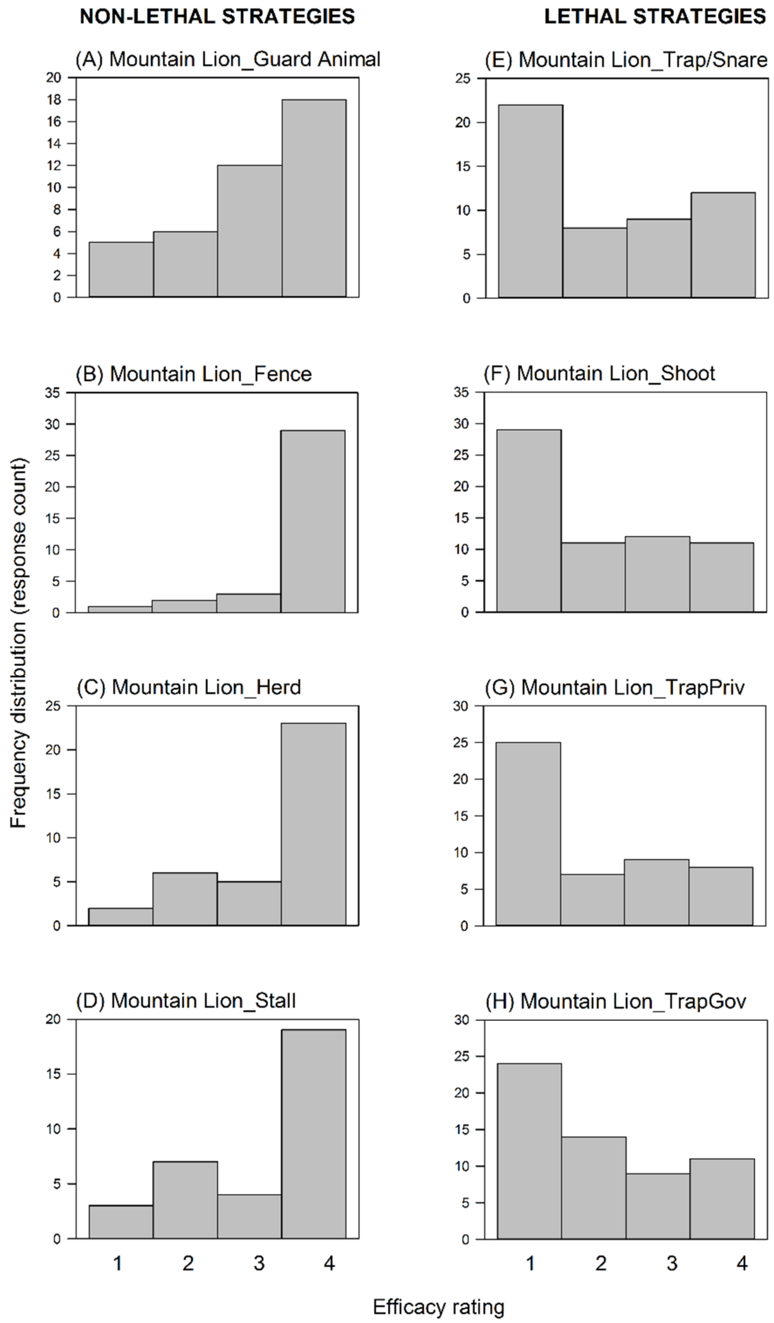

Figure A7. Histogram and frequency distribution of responses indicating efficacy of non-lethal (A – Guard Animals, B – Fencing, C – Herding, D – Stalling) and lethal (E – Trapping/Snaring, F – Shooting, G – Government Trapper, H – Private Trapper) mitigation strategies on mountain lions.

Appendix A. Histograms and frequency distributions

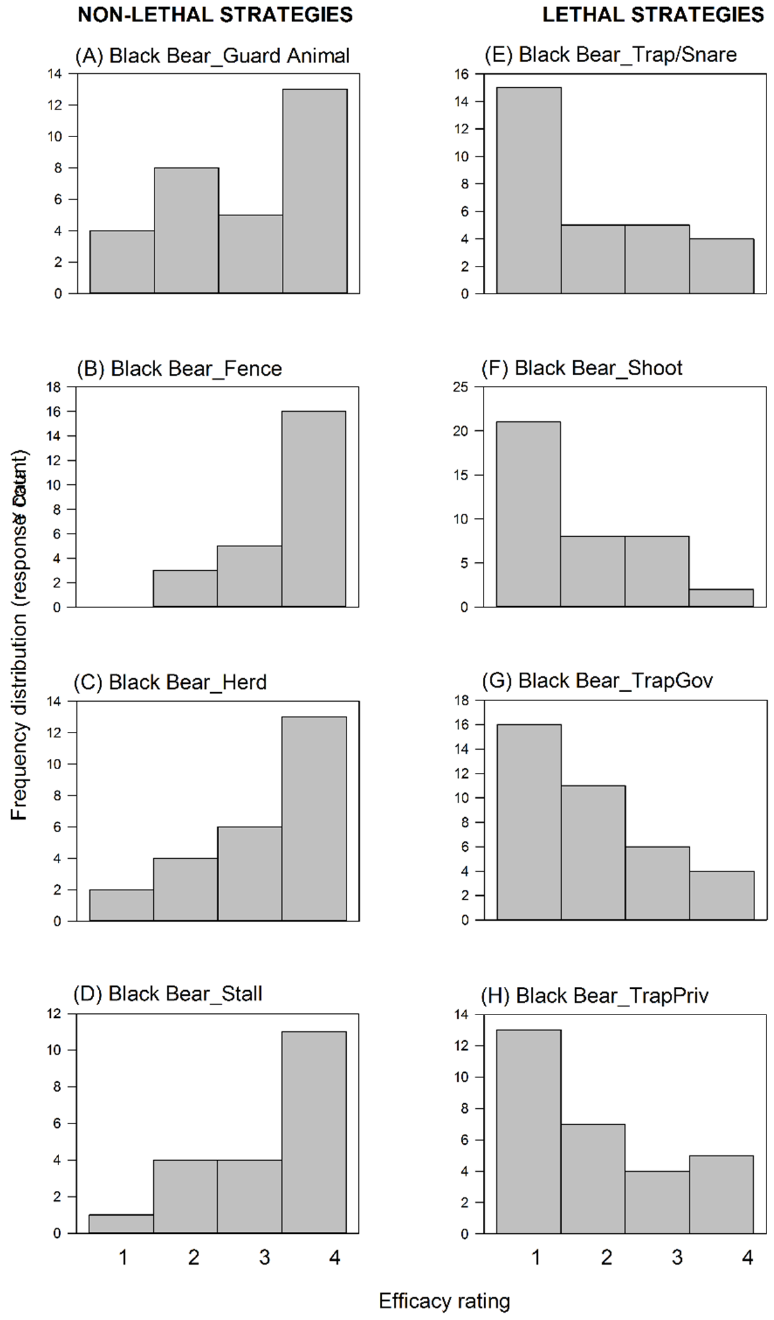

Figure A8. Histogram and frequency distribution of responses indicating efficacy of non-lethal (A – Guard Animals, B – Fencing, C – Herding, D – Stalling) and lethal (E – Trapping/Snaring, F – Shooting, G – Government Trapper, H – Private Trapper) mitigation strategies on black bears.

Appendix A. Histograms and frequency distributions

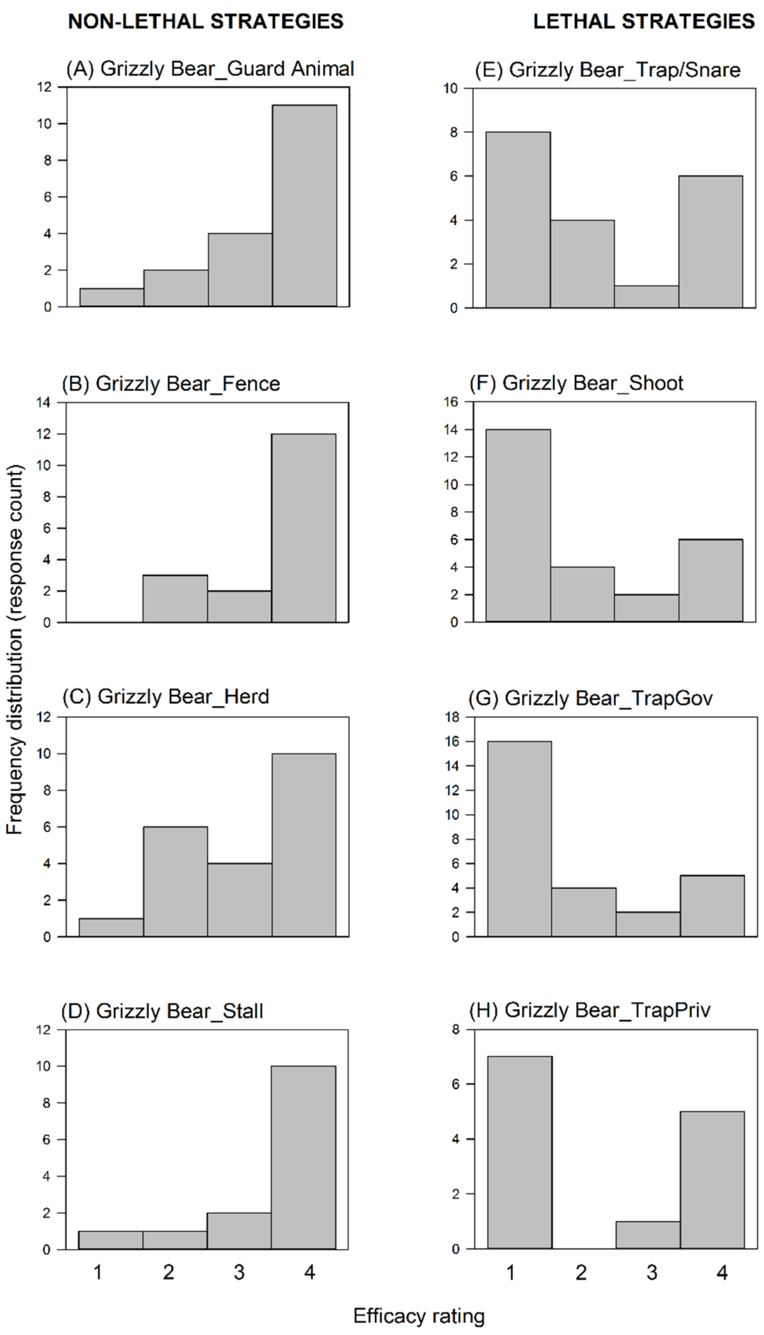

Figure A9. Histogram and frequency distribution of responses indicating efficacy of non-lethal (A – Guard Animals, B – Fencing, C – Herding, D – Stalling) and lethal (E – Trapping/Snaring, F – Shooting, G – Government Trapper, H – Private Trapper) mitigation strategies on grizzly bears.
