# Supplementary material for: Rancher-reported efficacy of lethal and non-lethal livestock predation mitigation strategies for a suite of carnivores
Source: Sci Rep. 2017 Oct 26;7:14105. doi: 10.1038/s41598-017-14462-1 (PMC5658346; doi:10.1038/s41598-017-14462-1)
Supplement: Supplementary file 2 — Appendix B. Efficacy rating sample size, median, mode, maximum, and minimum stratified by predator species, mitigation strategy, and livestock type. [file 41598_2017_14462_MOESM2_ESM.pdf]

Rancher-reported efficacy of lethal and non-lethal livestock predation mitigation strategies for a suite of carnivores

J.D. Scasta, B. Stam, and J. L. Windh

Appendix B. Efficacy rating sample size, median, mode, maximum, and mimimum stratified by predator species, mitigation strategy, and livestock type.

		Sample Size			Median			Mode			Maximum			Minimum		
**Predator Species**	**Mitigation Strategy**	**Both**	**Cattle**	**Sheep**	**Both**	**Cattle**	**Sheep**	**Both**	**Cattle**	**Sheep**	**Both**	**Cattle**	**Sheep**	**Both**	**Cattle**	**Sheep**
**Fox**	Guard Animals	24	7	6	2	2	2	2	1	1	3	4	4	1	1	1
	Fence	18	6	6	4	4	4	4	4	4	4	4	4	2	2	1
	Herding	17	7	5	2	3	4	2	4	4	4	4	4	1	1	3
	Stalling at Night	14	7	4	2	2	3	4	1	4	4	4	4	1	1	1
	Shooting	26	19	9	2	1	1	1	1	1	3	4	2	1	1	1
	Trapping/Snaring	27	14	9	2	1	1	1	1	1	4	4	2	1	1	1
	Trapper_Private	22	11	7	1	1	1	1	1	1	4	4	2	1	1	1
	Trapper_Government	29	13	8	1	2	1	1	2	1	3	4	3	1	1	1
**Dog**	Guard Animals	25	18	7	2	4	2	2	4	2	4	4	4	1	1	1
	Fence	14	19	6	4	4	4	4	4	4	4	4	4	1	1	1
	Herding	14	19	4	3	4	4	3	4	4	4	4	4	2	2	3
	Stalling at Night	16	20	4	3	4	3	4	4	4	4	4	4	1	1	1
	Shooting	29	72	8	1	1	1	1	1	1	4	3	2	1	1	1
	Trapping/Snaring	17	25	5	2	2	1	1	2	1	4	4	3	1	1	1
	Trapper_Private	15	22	5	1	2	1	1	4	1	4	4	2	1	1	1
	Trapper_Government	17	26	5	2	3	2	2	4	2	4	4	4	1	1	1
**Coyote**	Guard Animals	42	40	13	2	3	2	2	4	1	4	4	4	1	1	1
	Fence	28	39	8	3	4	4	4	4	4	4	4	4	1	1	1
	Herding	24	40	7	2	3	3	2	4	3	4	4	4	1	1	2
	Stalling at Night	26	40	7	2	3	2	1	4	1	4	4	4	1	1	1
	Shooting	40	129	12	1	1	2	1	1	1	3	4	3	1	1	1
	Trapping/Snaring	41	89	11	1	2	2	1	2	1	4	4	3	1	1	1
	Trapper_Private	32	79	9	1	1	1	1	1	1	4	4	2	1	1	1
	Trapper_Government	43	103	11	1	1	1	1	1	1	4	4	2	1	1	1
**Wolf**	Guard Animals	10	20	5	3	4	4	2	4	4	4	4	4	2	2	1
	Fence	8	22	5	4	4	4	4	4	4	4	4	4	2	2	2
	Herding	9	25	4	4	4	4	4	4	4	4	4	4	2	1	2
	Stalling at Night	8	21	2	4	4	4	4	4	4	4	4	4	2	1	4
	Shooting	10	38	5	1	1	1	1	1	1	4	4	3	1	1	1

		Sample Size			Median			Mode			Maximum			Minimum		
**Predator Species**	**Mitigation Strategy**	**Both**	**Cattle**	**Sheep**	**Both**	**Cattle**	**Sheep**	**Both**	**Cattle**	**Sheep**	**Both**	**Cattle**	**Sheep**	**Both**	**Cattle**	**Sheep**
Wolf cont...	Trapping/Snaring	9	25	4	2	2	2	1	1	3	4	4	3	1	1	1
	Trapper_Private	7	23	6	1	1	2	1	1	1	3	4	3	1	1	1
	Trapper_Government	10	45	7	2	1	1	1	1	1	3	4	3	1	1	1
**Bobcat**	Guard Animals	14	4	5	2	3	4	2	NA	4	4	4	4	1	1	1
	Fence	8	3	4	4	4	4	4	4	4	4	4	4	2	4	4
	Herding	9	3	4	2	4	4	4	4	4	4	4	4	1	3	4
	Stalling at Night	6	4	3	4	2	4	4	2	4	4	4	4	2	1	4
	Shooting	14	8	4	2	1	2	1	1	1	4	2	4	1	1	1
	Trapping/Snaring	16	8	7	1	1	1	1	1	1	4	2	3	1	1	1
	Trapper_Private	14	7	7	2	1	1	1	1	1	4	2	2	1	1	1
	Trapper_Government	17	6	4	1	1	2	1	1	2	4	2	4	1	1	1
**Lion**	Guard Animals	20	16	5	3	4	2	3	4	4	4	4	4	1	3	1
	Fence	14	18	3	4	4	4	4	4	4	4	4	4	1	2	4
	Herding	15	17	4	3	4	4	4	4	4	4	4	4	2	1	2
	Stalling at Night	12	18	3	4	4	4	4	4	4	4	4	4	2	1	2
	Shooting	19	40	4	2	2	2	1	1	3	4	4	3	1	1	1
	Trapping/Snaring	21	26	4	2	2	2	1	1	1	4	4	3	1	1	1
	Trapper_Private	22	23	4	1	2	2	1	1	3	4	4	3	1	1	1
	Trapper_Government	20	33	5	2	2	1	1	1	1	4	4	3	1	1	1
**Black Bear**	Guard Animals	19	6	5	3	4	2	2	4	4	4	4	4	1	3	1
	Fence	16	6	2	4	4	4	4	4	4	4	4	4	2	3	4
	Herding	16	7	2	3	4	4	4	4	4	4	4	4	1	3	4
	Stalling at Night	12	6	2	4	4	3	4	4	NA	4	4	4	2	1	2
	Shooting	19	16	4	2	1	2	1	1	3	4	4	3	1	1	1
	Trapping/Snaring	18	8	3	2	1	3	1	1	3	4	4	3	1	1	1
	Trapper_Private	15	10	4	2	1	2	1	1	2	4	4	3	1	1	1
	Trapper_Government	17	16	4	2	2	2	1	1	2	4	4	3	1	1	1
**Grizzly Bear**	Guard Animals	7	10	1	3	4	1	3	4	NA	4	4	1	2	3	1
	Fence	6	11	0	4	4	NA	4	4	NA	4	4	NA	2	2	NA
	Herding	7	14	0	3	4	NA	2	4	NA	4	4	NA	2	1	NA
	Stalling at Night	5	9	0	4	4	NA	4	4	NA	4	4	NA	2	1	NA

Predator Species	Mitigation Strategy	Sample Size			Median			Mode			Maximum			Minimum		
		Both	Cattle	Sheep	Both	Cattle	Sheep	Both	Cattle	Sheep	Both	Cattle	Sheep	Both	Cattle	Sheep
	Shooting	8	18	0	2	1	NA	1	1	NA	4	4	NA	1	1	NA
	Trapping/Snaring	7	12	0	2	2	NA	1	1	NA	4	4	NA	1	1	NA
Grizzly Bear cont...	Trapper_Private	6	7	0	1	4	NA	1	4	NA	4	4	NA	1	1	NA
	Trapper_Government	7	20	0	1	1	NA	1	1	NA	4	4	NA	1	1	NA
**Birds**	Guard Animals	28	24	9	3	4	4	4	4	4	4	4	4	1	1	1
	Fence	21	23	6	4	4	4	4	4	4	4	4	4	4	4	4
	Herding	23	25	6	4	4	4	4	4	4	4	4	4	2	2	4
	Stalling at Night	21	24	5	4	4	4	4	4	4	4	4	4	1	1	2
	Shooting	20	49	6	4	2	3	4	1	4	4	4	4	1	1	1
	Trapping/Snaring	21	25	6	4	4	3	4	4	4	4	4	4	1	1	1
	Trapper_Private	20	24	6	4	4	4	4	4	4	4	4	4	1	1	1
	Trapper_Government	26	35	7	3	3	4	4	4	4	4	4	4	1	1	1
